# Study on the Performance Enhancement Mechanism of Basalt Fiber-Reinforced Hydraulic Concrete in Ship Lock Galleries

**DOI:** 10.3390/ma19071333

**Published:** 2026-03-27

**Authors:** Benkun Lu, Jie Chen, Shuncheng Xiang, Zhe Peng, Changyu Liu, Linna Li

**Affiliations:** 1 School of Hydraulic and Ocean Engineering, Changsha University of Science & Technology, Changsha 410114, China; 2 Hunan Provincial Department of Transportation, Changsha 410000, China; 3 Key Laboratory of Water-Sediment Sciences and Water Disaster Prevention of Hunan Province, Changsha 410114, China; 4 Hunan Provincial Water Transport Construction & Investment Group Co., Ltd., Changsha 410029, China

**Keywords:** basalt fiber, ship lock, orthogonal experiment, crack resistance, freeze–thaw resistance, impermeability, abrasion–erosion resistance

## Abstract

This study investigated the enhancement mechanisms and optimal mix proportion of basalt fiber (BF) in concrete for ship lock galleries. It focused on improving crack resistance, freeze–thaw resistance, impermeability, and abrasion–erosion resistance under complex hydraulic environments. Single-factor tests first determined the reasonable parameter ranges, which were subsequently used in a three-factor, four-level orthogonal experiment to analyze the effects of the water-to-binder ratio, fiber content, and fiber length on concrete’s mechanical properties. Range analysis of the orthogonal experiment indicated that the water-to-binder ratio was the most dominant factor (R = 57.4), followed by fiber content. Based on this, further durability tests were conducted, including ring restraint cracking, impermeability, freeze–thaw resistance, and abrasion–erosion resistance. Multi-objective optimization was performed using full factorial experiments and a comprehensive performance evaluation system. The final optimal mix proportion was determined as: a water-to-binder ratio of 0.35, a fiber content of 0.2%, and a fiber length of 12 mm. With this mix, the concrete’s ring cracking time was extended by 69.9%, the relative dynamic elastic modulus retention reached 73.0% after 100 freeze–thaw cycles, the relative permeability coefficient was 1.04 × 10^−6^ cm/h, and the abrasion–erosion resistance strength increased to 7.05 h·m^2^/kg, which achieved an optimal synergy among the mechanical properties, key durability indicators, and their workability. Mechanism analysis revealed that BF formed a three-dimensional, randomly distributed fiber network that comprehensively enhanced concrete performance through multi-scale mechanisms, including bridging, pore refinement, and energy dissipation. This research has provided systematic experimental evidence and mix proportion support for the durability design and engineering application of BF concrete in ship lock galleries.

## 1. Introduction

As the core water conveyance structure of navigation hubs, ship lock galleries are exposed to harsh environments featuring high-speed sediment-laden flow [1,2], wet–dry cycles, and freeze–thaw alternation for extended periods of time. Their concrete flow surfaces commonly face aggregate abrasion and spalling caused by high-velocity water flow [3], as well as freeze–thaw cycle damage induced by temperature changes. These factors synergistically accelerate the deterioration of the structure’s mechanical and durability performance, which can even lead to large-area concrete detachment and seriously threaten the safe operation and long-term durability of the ship lock [4].

Basalt fiber (BF) is an eco-friendly inorganic fiber produced by melting and drawing natural basalt ore. It has attracted considerable attention in civil engineering for crack resistance and reinforcement due to its high strength, excellent chemical resistance, and sustainable characteristics. When incorporated into concrete, BF can form a three-dimensional network structure that bridges micro-cracks and enhances material toughness [5]. These factors are not only governed by parameters, such as fiber content and length, but are also profoundly influenced by the water-to-binder ratio.

Although the application of BF in conventional structures such as pavements and bridge decks has become relatively mature [6,7], research specifically addressing the multi-field environment of ship lock galleries remains severely insufficient. Existing studies exhibit two main limitations as follows.

First, there is a methodological gap in current optimization approaches. Nawaf et al. [5] recommended 0.2–0.5% for comprehensive mechanical properties, Guo Weiwei [8] found the optimal content for abrasion resistance to be only 0.1%, while Hao et al. [9] suggested a range of 0.1–0.5% under coupled salt–frost and wet–dry environments. These discrepancies suggest that fiber reinforcement is not solely governed by content but is closely tied to the properties of the cement matrix. Research by Li et al. [10] has provided a critical clue in this regard, as they found that a lower water-to-cement ratio significantly increased fiber pull-out load, indicating that the water-to-binder ratio determined the stress transfer efficiency between fibers and the matrix by influencing the compactness of the interfacial transition zone (ITZ).

Second, existing research critically lacks engineering specificity. Unlike conventional structures, ship lock galleries are subjected to the coupled effects of high-velocity abrasion, penetration pressure, and freeze–thaw cycles—a complexity rarely replicated in current studies. Hao et al. [9] investigated the coupling of salt–frost and wet–dry cycles but did not incorporate abrasion factors; meanwhile, Zhang et al. [11] studied seawater erosion but focused on the flexural behavior of beam specimens rather than lining structures. Furthermore, the existing literature overwhelmingly emphasizes conventional mechanical properties such as compressive strength and flexural strength, while systematic evaluations of key durability indicators for gallery concrete under coupled deterioration conditions remain scarce.

To address these research gaps, this study investigates basalt fiber incorporation in ship lock gallery concrete, aiming to systematically explore its influence on crack resistance, abrasion–erosion resistance, impermeability, and freeze–thaw resistance, as well as its underlying mechanisms. A progressive experimental approach is adopted, integrating single-factor tests; three-factor, four-level orthogonal experiments; and full factorial experiments. Single-factor tests are first conducted to determine the reasonable ranges of key parameters (water-to-binder ratio, fiber content, and fiber length). Based on these ranges, an orthogonal experiment has been designed to analyze the primary and secondary effects of the three factors on mechanical performance and to identify the theoretically optimal combination. Subsequently, with the optimal water-to-binder ratio fixed, a full factorial experiment has been performed to quantify the interaction between fiber content and length, and a weighted comprehensive evaluation system has been established to determine the globally optimal mix proportion for ship lock gallery concrete. Systematic durability testing and mechanistic analysis of the optimized groups is then conducted to provide solid experimental evidence and theoretical support for enhancing the long-term durability of ship lock gallery concrete in complex hydraulic environments.

## 2. Materials and Methods

### 2.1. Raw Materials

Cement: Ordinary Portland cement (P·O 42.5, Conch Cement Co., Ltd., Wuhu, China) was used. Its 3-day compressive strength was no less than 20 MPa and the 28-day compressive strength was no less than 42.5 MPa, while the initial setting time was 120 min and the final setting time was 360 min. The loss on ignition was less than 5.0%, the soundness was qualified, and the standard consistency water requirement met specifications [12].

Fine Aggregate: River sand was adopted with a fineness modulus of 2.7, an apparent density of 2650 kg/m^3^, a bulk density of 1580 kg/m^3^, and a void content of 42%, exhibiting a hard texture, good grading, and cleanliness [13].

Coarse Aggregate: Crushed stone was selected with a particle size range of 5–25 mm, an apparent density of 2720 kg/m^3^, a bulk density of 1620 kg/m^3^, and a void content of 42%. The content of flaky and elongated particles was below 15%, and the crushing index was below 10% [13].

Basalt Fiber: The fiber (Haining Anjie Co., Ltd., Haining, China) were used with a diameter of 15 µm, the length ranged from 3 mm to 25 mm, the tensile strength ranged from 3000 to 4800 MPa, the elastic modulus ranged from 91 to 110 GPa, and the density was 2650 kg/m^3^ [14].

### 2.2. Single-Factor Experiments

For all mechanical and durability tests, at least three replicate specimens were tested for each mix, and the results were reported as averages [15,16].

Prior to the orthogonal experiment, reasonable value ranges for key influencing factors (fiber content, length, and water-to-binder ratio) were determined through single-factor experiments, and the variables were controlled individually to determine their reasonable ranges based on the literature and engineering practices. The experimental scheme is shown in Table 1.

### 2.3. Orthogonal Experiment

Reasonable level ranges for each factor were determined based on the results of the single-factor tests. The water-to-binder ratio range of 0.35–0.45 was selected to ensure both mechanical performance and constructability (slump > 50 mm). The fiber content was selected from 0.1% to 0.4% because this range covered the complete interval from performance improvement to degradation, avoiding the severe workability deterioration and agglomeration risk at 0.5%. The fiber length was selected from 6 mm to 18 mm because 3 mm fibers had a weak enhancement effect, while 24 mm fibers were prone to introducing agglomeration defects; consequently, 6–18 mm was the effective range for ensuring dispersibility and enhancement effect.

This paper employed an L_16_(4^3^) orthogonal array with three factors and four levels for experimental design, using fiber content, fiber length, and water-to-binder ratio as variables to study their influence on the mechanical properties of basalt fiber-reinforced concrete. The L_16_(4^3^) orthogonal array was selected for its two key characteristics: a balanced dispersion, which ensured that all levels of each factor were uniformly distributed across the experiments; and neat comparability, which guaranteed that any two combinations of different factor levels appeared an equal number of times. These properties enabled comprehensive information to be obtained with a reduced number of experimental runs—only 16 groups compared to 64 groups required for a full factorial design (4^3^)—while still allowing the accurate analysis of the primary and secondary effects of each factor, as well as the identification of optimal level combinations [17]. The experiments were arranged according to the orthogonal array, resulting in a total of 16 experimental groups, each corresponding to a different combination of factor levels, as detailed in Table 2.

Testing was conducted in accordance with the SL/T 352-2020, Test code for hydraulic concrete [18]. Compressive strength and splitting tensile strength tests were performed using 100 mm × 100 mm × 100 mm cubic specimens and tested with a microcomputer-controlled stiffness testing machine (Model: WHY-3000, Shanghai Hualong Test Instruments Co., Ltd., Shanghai, China), with a maximum test force of 3000 kN. Four-point bending tests were conducted using 100 mm × 100 mm × 400 mm prismatic specimens and tested with a servo-controlled universal testing machine (WAN-1000, Shanghai Hualong Test Instruments Co., Ltd., Shanghai, China) with a maximum test force of 1000 kN and an accuracy of Class 0.5. All test results were taken as the average values of three replicate specimens.

Based on the comprehensive index analysis of the three-factor, four-level orthogonal array, the following key groups were selected for systematic durability performance testing:

1. Reference Group (JZ): A water-to-binder ratio of 0.35 without any fiber addition.
2. Comprehensive Performance Optimal Group: The combination with the highest comprehensive score from the orthogonal experiment.
3. Theoretical Optimal Group: The theoretical combination determined through range analysis based on the optimal level of each factor.
4. Peak Performance Groups: The group with the highest measured compressive strength, the group with the highest splitting tensile strength, and the group with the highest flexural strength in the orthogonal experiment.

### 2.4. Durability Performance Tests

- Ring Restraint Cracking Test

The ring restraint cracking test method refers to the principle of ASTM C1581, Standard Test Method for Determining Age at Cracking and Induced Tensile Stress Characteristics of Mortar and Concrete under Restrained Shrinkage [19,20]. Its core lies in using an inner steel ring to apply circumferential restraint to an external annular concrete specimen, which simulates the tensile stress state in concrete structures during its early age as shrinkage can be restrained by internal reinforcement and boundaries, thereby inducing and quantifying the tendency for plastic shrinkage cracking [20].

In this study, cement paste rather than concrete was used for the ring test. This choice was made for two primary reasons: (1) to accelerate the cracking process—cement paste exhibited higher shrinkage than concrete, allowing cracks to be initiated within a shorter time frame and thus, improved test efficiency when screening multiple fiber parameters; and (2) to eliminate the interference of coarse aggregates on fiber distribution and shrinkage behavior, thereby focusing the evaluation purely on the fiber–matrix interaction [19]. The use of paste also facilitated subsequent microscopic observations (e.g., SEM) of fiber bridging and interfacial transition zone morphology.

However, it must be emphasized that the paste system differed from concrete in its aggregate interaction, shrinkage mechanism, and fiber distribution; consequently, the cracking times measured in this test cannot be directly equated to the absolute performance of concrete. The results are intended for qualitative comparisons of the relative effectiveness of different fiber parameters in inhibiting early-age plastic shrinkage cracks, providing trend guidance for engineering material selection rather than serving for quantitative prediction. The final validation of the optimal mix proportion for concrete was performed through concrete-level durability tests as described in the following sections (impermeability, freeze–thaw, and abrasion–erosion resistance).

**Figure 1:**
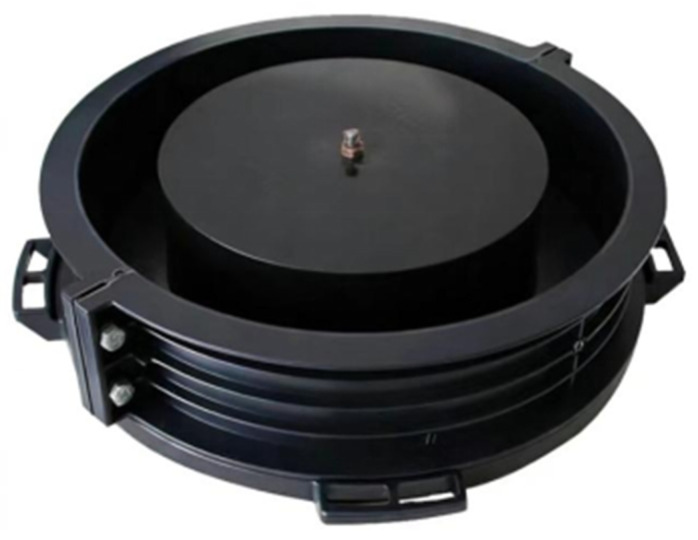
Experimental mold.

The dimensions of the ring test specimens were as follows: outer diameter × inner diameter × height = 425 mm × 305 mm × 100 mm (Figure 1). The cracking time and crack width were then measured. The cracking time was recorded as the time of first crack initiation, and the maximum crack width was measured using a crack width gauge 24 h after crack initiation [20].

- Abrasion–Erosion Resistance Test

To simulate the scouring and wearing effects of high-speed sediment-laden water flow in ship lock galleries, the underwater steel ball method was selected for the test and followed SL/T 352-2020, Test code for hydraulic concrete [18]. A rotary underwater steel ball abrasion machine (GB-YX, Xi’an Yaxing Civil Engineering Instrument Co., Ltd., Xi’an, China) with a rotational speed of 1200 r/min was used. The specimens were flat cylinders with a diameter of 300 mm and a height of 100 mm. The specimens were subjected to 72 h of continuous abrasion testing, after which the mass loss was measured and the abrasion–erosion resistance strength calculated [21].

- Impermeability Test

The impermeability test employed the relative permeability method and followed SL/T 352-2020, Test code for hydraulic concrete [18]. Truncated cone-shaped specimens with a top diameter of 175 mm, a bottom diameter of 185 mm, and a height of 150 mm were used. Specimens were cured to the specified age, their sides were coated with sealing material, and they were then placed into a permeameter. Pressure was applied at 0.8 MPa in one increment and maintained for 24 h. The time when a stable pressure was reached was recorded as the test start time. During the constant pressure period, the water seepage condition on the specimen end face was observed at all times. If water seepage appeared on the specimen end face, the test was stopped immediately, and the time of the water’s appearance was recorded. If no water seepage appeared on the specimen end face after 24 h of pressurization, the test was stopped, and the specimen was removed.

The specimen was then placed in a pressure testing machine and split into two halves along the longitudinal section. After observing the water penetration, the outline of water penetration was traced, and the water penetration height was measured.

- Freeze–Thaw Cycle Test

The freeze–thaw cycle test was conducted following SL/T 352-2020, Test code for hydraulic concrete [18]. Prism specimens of 100 mm × 100 mm × 400 mm were used. A fully automatic concrete rapid freeze–thaw testing machine (TDR-28, Tianjin Gangyuan Test Instrument Factory, Tianjin, China) was employed. An intermediate inspection, involving weighing and measuring natural frequency, was performed every 25 freeze–thaw cycles, after which the specimen was then returned to the freeze–thaw testing machine to continue the test. Finally, the relative dynamic elastic modulus and mass loss rate were calculated.

### 2.5. Full Factorial Experiment Design

Based on the orthogonal experiment results, a water-to-binder ratio of 0.35 was established as the fixed parameter. A full factorial experiment was then designed to quantify interactions between fiber content (0.20%, 0.25%, 0.30%) and fiber length (9 mm, 12 mm, 15 mm) at this optimal ratio, using comprehensive durability indicators as the evaluation criteria.

According to the full factorial design method, a total of nine experimental mix proportions were formed. All groups underwent four key durability tests: ring restraint cracking, impermeability, freeze–thaw resistance, and abrasion–erosion resistance (following the procedures in Section 2.3). A weighted comprehensive performance evaluation system was established based on the actual service requirements of ship lock gallery concrete to determine the globally optimal mix proportion.

### 2.6. Microstructural Analysis

To reveal the reinforcement mechanism of basalt fiber on concrete performance, scanning electron microscopy (SEM) was employed to observe the microstructure of basalt fiber-reinforced concrete [22].

After the completion of macroscopic mechanical tests, representative concrete fragments were selected from each group of specimens for microstructural analysis. A ZEISS field emission scanning electron microscope (EVO 18, Carl Zeiss AG, Oberkochen, Germany) was used to observe the microstructure of the fiber-reinforced concrete, with the following key technical parameters: a secondary electron image resolution of 3.0 nm at 30 kV; an accelerating voltage range of 0.2–30 kV; and a magnification range of 5–1,000,000×. By observing the microstructure at magnifications of 100× and 500×, particular attention could be paid to the dispersion state of fibers in the matrix, the morphology of the fiber–cement matrix interfacial transition zone (ITZ), fiber bridging at micro-cracks, and the pore structure characteristics of the matrix, thereby elucidating the reinforcement mechanism of basalt fiber on concrete performance.

## 3. Results

### 3.1. Single-Factor Test Results and Analysis

#### 3.1.1. Effect of Water-to-Binder Ratio

**Figure 2:**
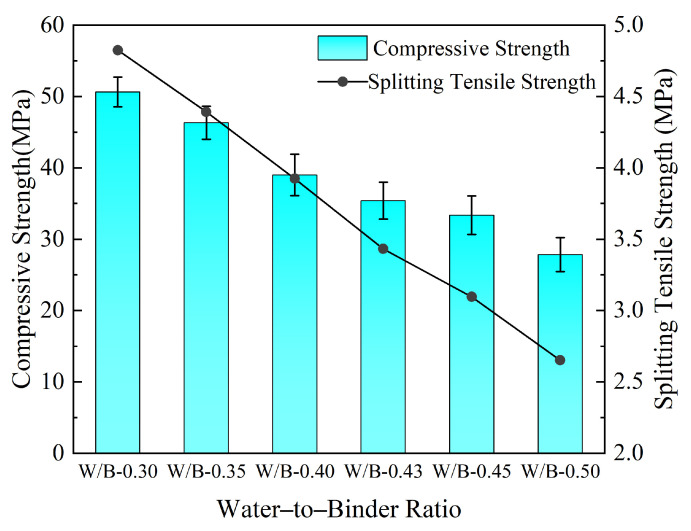
Effect of water-to-binder ratio on concrete compressive strength and splitting tensile strength.

Figure 2 presents the variation curves of 28-days compressive strength and splitting tensile strength of concrete under different water-to-binder ratios.

As the water-to-binder ratio decreased from 0.50 to 0.30, the compressive strength continuously increased, reaching a maximum value of 50.64 MPa at 0.30; the splitting tensile strength increased correspondingly, peaking at 3.86 MPa at 0.30. This result aligned with the findings by Arianti et al. [23].

**Figure 3:**
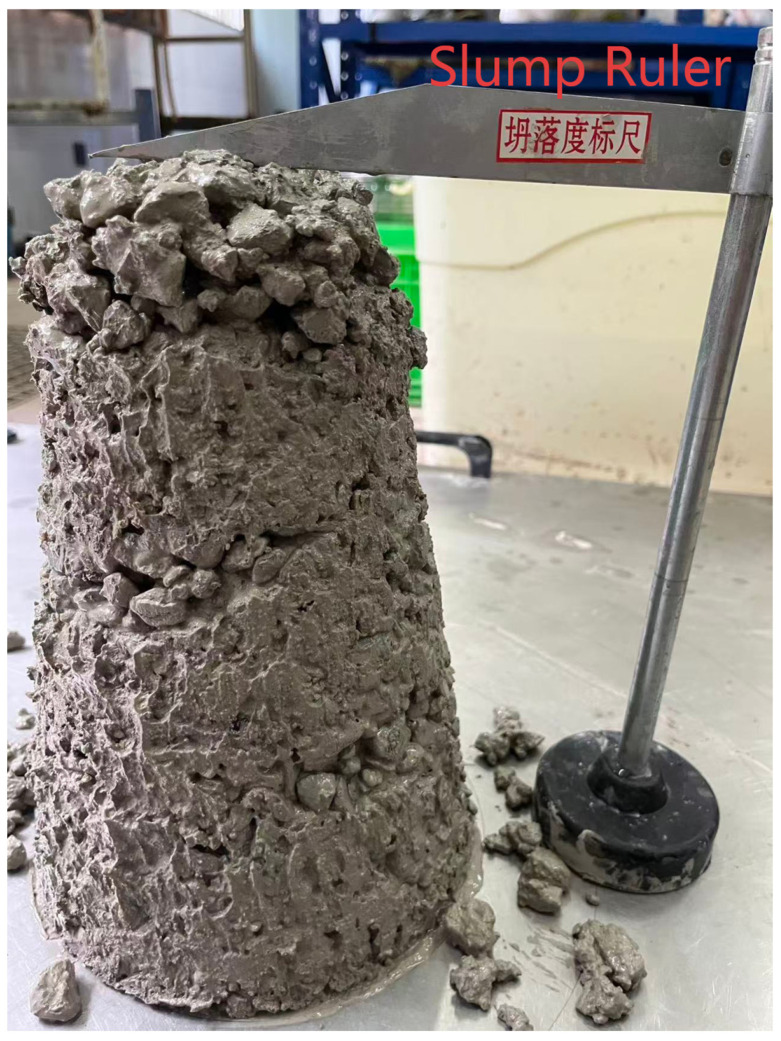
Slump test result for concrete with water-to-binder ratio of 0.30, showing a slump value of 32 mm and indicating poor workability.

However, when the water-to-binder ratio was as low as 0.30, the water absorption of the cementitious materials increased, leading to insufficient free water and a sharp drop in slump to 32 mm (Figure 3). This severely deteriorated workability, rendering it unsuitable for engineering applications in ship lock gallery structures that require good casting compactness. Therefore, in the orthogonal experiment, 0.35 was used as the starting point for the low water-to-binder ratio level to achieve a balance between high strength and constructability.

#### 3.1.2. Effect of Fiber Content

**Figure 4:**
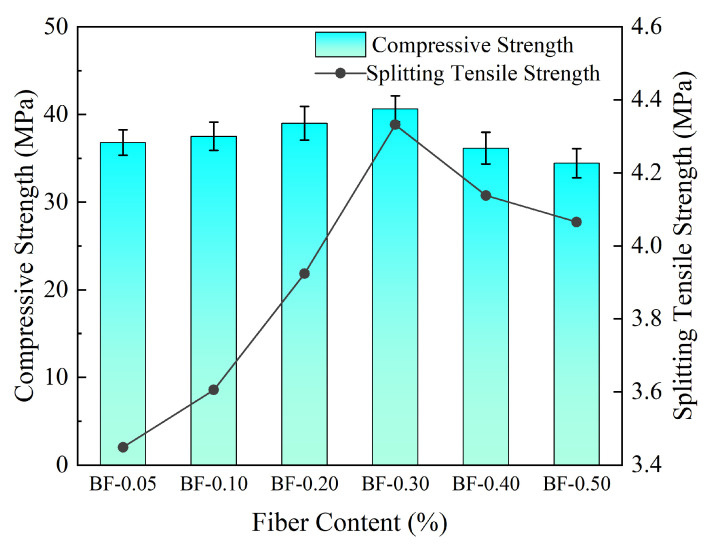
Effect of fiber content on concrete compressive strength and splitting tensile strength.

Figure 4 illustrates the variation patterns of compressive strength and splitting tensile strength of concrete under different fiber contents.

There exists an optimal range for the content of basalt fiber in concrete. As the fiber content increased from 0.05% to 0.2%, the compressive strength increased from 36.80 MPa to 40.64 MPa, and the splitting tensile strength increased from 3.45 MPa to 4.33 MPa. When the content further increased to 0.5%, the compressive strength decreased to 34.44 MPa, and the splitting tensile strength decreased to 4.06 MPa. Based on this, the fiber content range for the orthogonal experiment was determined to be 0.1–0.4%.

#### 3.1.3. Effect of Fiber Length

**Figure 5:**
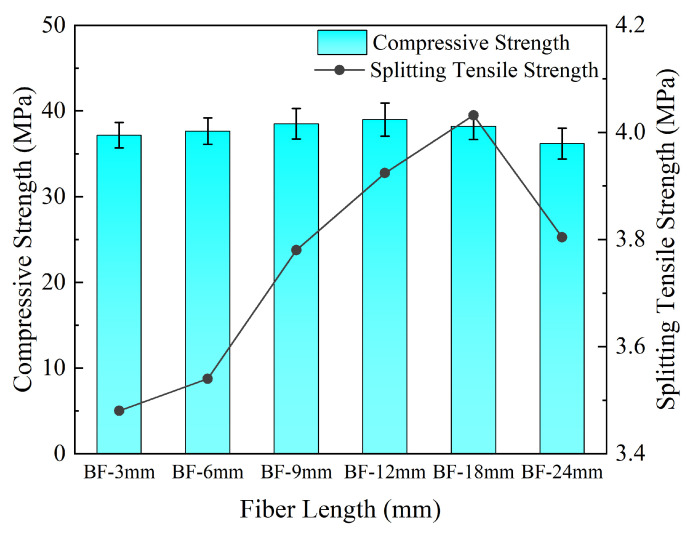
Effect of fiber length on concrete compressive strength and splitting tensile strength.

Figure 5 shows the test results of compressive strength and splitting tensile strength of concrete under different fiber lengths.

When the fiber length increased from 3 mm to 12 mm, the compressive strength continuously increased from 37.16 MPa to 39.02 MPa, and the splitting tensile strength increased from 3.48 MPa to 3.92 MPa. When the length further increased to 18 mm, the compressive strength decreased to 38.2 MPa, while the splitting tensile strength increased to 4.03 MPa. However, when the fiber length was ≥18 mm, fiber entanglement and agglomeration were observed during the mixing process; therefore, the fiber length range for the orthogonal experiment was determined to be 6–18 mm.

### 3.2. Orthogonal Experiment Results Analysis

To comprehensively evaluate the influence of each factor on mechanical performance, a comprehensive index was derived by processing the three indicators together, as listed in Table 3. The specific method was as follows: first, the maximum value of each indicator was set to 100 with a minimum of 0, while other measured values were converted to a percentage scale using range normalization; then, a weighted comprehensive score was calculated based on the assigned weights.

The determination of weight coefficients followed two fundamental principles: (1) the structural stress characteristics of ship lock gallery concrete; and (2) the synergistic roles of different mechanical indicators in fiber-reinforced composites.

As the lining of a ship lock gallery is a water conveyance structure that primarily bears circumferential tensile stress induced by internal water pressure, indicators characterizing the material’s resistance to bending and direct tension were more critical than compressive strength when evaluating its comprehensive mechanical performance [24]. Accordingly, flexural strength, which can reflect a component’s resistance to bending tensile stress, was assigned the highest weight of 40%. The splitting tensile strength, which characterizes direct tensile performance, was assigned a weight of 30% as a complementary indicator. The compressive strength was assigned a weight of 30% to reflect its foundational contribution to overall performance.

This weighting method, which combined range normalization with linear weighted scoring, has been widely used in multi-criteria decision-making for material performance evaluation.

Range analysis (Table 3) indicated that the water-to-binder ratio had the greatest influence on comprehensive performance (R = 57.4), followed by fiber content (R = 46.8), while fiber length had the smallest influence (R = 11.4). The optimal levels for each factor were: a water-to-binder ratio of 0.35 (K1 = 82.7), a fiber content of 0.2% (K2 = 68.7), and a fiber length of 12 mm (K3 = 53.9). 

As shown in Table 4, regarding compressive strength, splitting tensile strength, flexural strength, and the comprehensive index, both the water-to-binder ratio and fiber content exhibited highly significant effects (*p* < 0.01). The fiber length showed no significant effect on any of the evaluated indicators, which was consistent with the range analysis results where fiber length had the smallest range value (R = 11.4).

Based on the comprehensive index analysis of the orthogonal experiment (Table 3), the following four representative mix proportions were selected for systematic durability performance testing to reveal performance differences under different optimization objectives:

1. Reference Group (JZ): water-to-binder ratio 0.35 with no fiber added—this served as the baseline for performance comparison.
2. Group C1: water-to-binder ratio 0.35, fiber content 0.2%, and fiber length 9 mm—this combination had the highest measured compressive strength in the orthogonal experiment.
3. Group C2: water-to-binder ratio 0.35, fiber content 0.2%, and fiber length 12 mm—this combination was the theoretical optimal group determined through range analysis (water-to-binder ratio level 1, content level 2, length level 3), representing the global optimal solution based on statistical methods.
4. Group C3: water-to-binder ratio 0.35, fiber content 0.3%, and fiber length 12 mm—this combination had the highest flexural strength and splitting tensile strength among all 16 groups and also achieved the highest comprehensive score in the orthogonal experiment, representing the optimal comprehensive mechanical performance.

### 3.3. Ring Restraint Cracking Test Analysis

In this section, to correspond with the paste test system, the test groups for the ring restraint cracking test have been denoted as JZ′, C1′, C2′, and C3′. Their water-to-binder ratio was the same as that of the concrete test groups, JZ, C1, C2, and C3 (0.35), but they contained no coarse or fine aggregates. This allowed for the crack resistance effect of the water-to-binder ratio and fibers to be examined purely at the paste’s scale.

The test results shown in Table 5 indicate that incorporating basalt fibers can significantly enhance the crack resistance of cement paste. The initial cracking time for the reference group JZ′ was 40.8 h, while the cracking times for the groups with added fiber were generally extended to over 50 h. The cracking time for group C1′ (9 mm) was extended by 60.8%, and for group C2′ (12 mm) by 69.9%. In terms of limiting crack width, groups C2′ and C3′ reduced the maximum crack width by 68% and 100%, respectively [25,26].

### 3.4. Impermeability Test Results Analysis

A lower relative permeability coefficient indicated superior impermeability of the concrete. As shown in Table 6, impermeability ranking began with C2 (best), followed by C3, C1, and JZ. The relative permeability coefficient of group C2 was 26.2% lower than that of the reference group.

### 3.5. Freeze–Thaw Cycle Test Results Analysis

**Figure 6:**
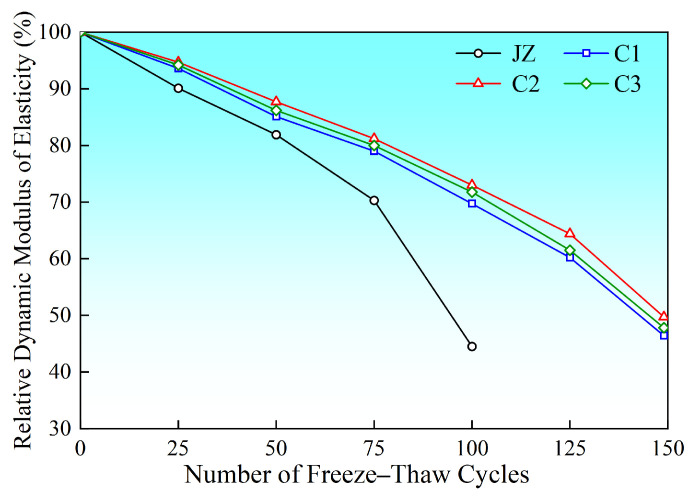
Relationship curve between relative dynamic elastic modulus and number of freeze–thaw cycles.

**Figure 7:**
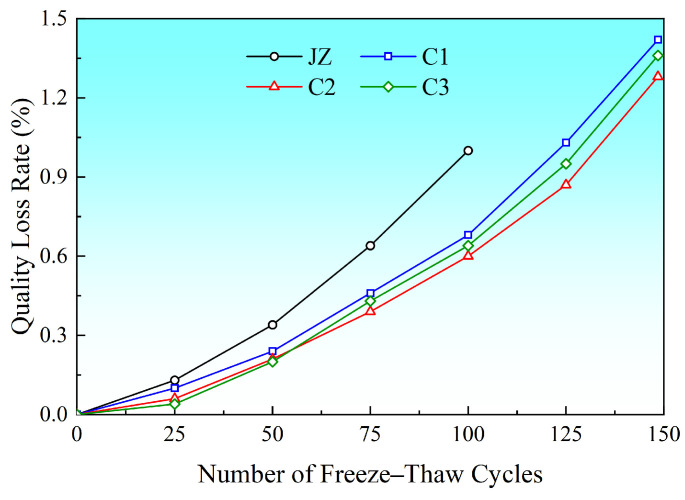
Relationship curve between mass loss rate and number of freeze–thaw cycles.

After 150 freeze–thaw cycles, the basalt fiber-reinforced concrete exhibited excellent freeze–thaw resistance durability (Figure 6 and Figure 7). However, for effective comparison, we have focused on the performance at the key milestone of 100 cycles. As shown in the figures, the relative dynamic elastic modulus of the reference group (JZ) had dropped to 44.5% after 100 freeze–thaw cycles. All specimens with added fibers (C1–C3) performed significantly better than the reference group, with retention rates reaching 69.7%, 73.0%, and 71.8%, respectively. It was only after 150 cycles that the relative dynamic elastic modulus of the fiber groups dropped below 60%. This experimental result was highly consistent with the findings reported in the literature by Tang Huang [27].

### 3.6. Abrasion–Erosion Resistance Test Results Analysis

**Figure 8:**
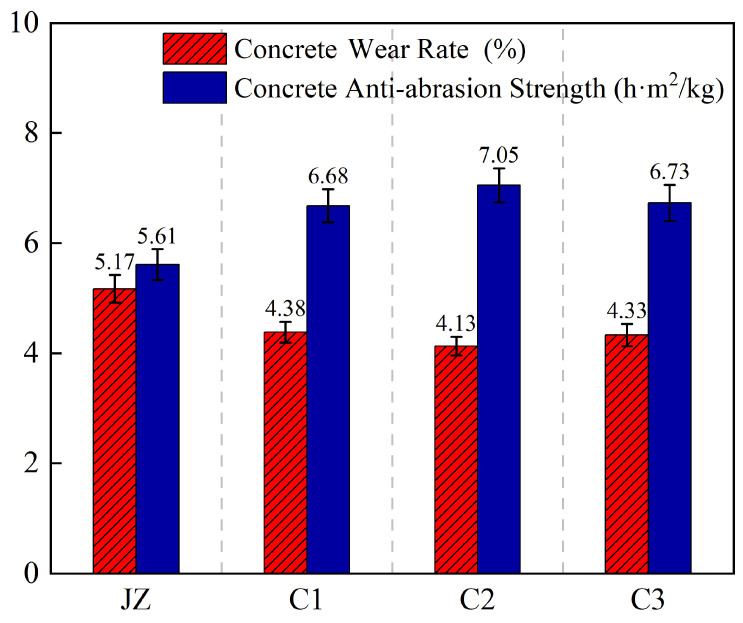
Abrasion–erosion resistance test results.

Figure 8 presents the 72 h abrasion–erosion resistance strength test results for each group of concrete. The incorporation of basalt fibers significantly enhanced the abrasion–erosion resistance of hydraulic concrete in high-velocity water flow environments. The test results showed that the abrasion–erosion resistance strength of the reference group (JZ, no fiber) was 5.61 h·m^2^/kg. After fiber incorporation, the performance of all groups improved noticeably: group C1 increased to 6.68 h·m^2^/kg, an improvement of 19.07%; group C2 performed best and reached 7.05 h·m^2^/kg, an improvement of 25.67%; and group C3 had an abrasion–erosion resistance strength of 6.73 h·m^2^/kg, an improvement of 19.96% compared to the reference group.

### 3.7. Fiber Parameter Optimization and Optimal Mix Proportion Determination

The test results showed that group C3 with a fiber content of 0.3% presented a unique performance combination: it had the best impermeability and the smallest measured crack width in the ring restraint cracking test; however, its mechanical indicators such as compressive strength, splitting tensile strength, and initial cracking time were inferior to those of the comprehensively optimal group C2. This contradiction highlighted the limitations of single performance indicator evaluation. To precisely determine the globally optimal mix proportion for the ship lock gallery environment, this section employed comprehensive scoring and range analysis based on the full factorial experiment data from Section 2 for multi-objective optimization.

#### 3.7.1. Establishment of the Comprehensive Performance Evaluation System

To quantitatively compare the results of multiple test groups, a comprehensive performance evaluation system was established in this study. The specific steps were as follows.

The test results of the nine groups on four key durability indicators—ring restraint cracking (initial cracking time, maximum crack width); impermeability (relative permeability coefficient); freeze–thaw resistance (relative dynamic elastic modulus after 100 freeze–thaw cycles); and abrasion–erosion resistance (abrasion–erosion resistance strength)—were normalized. All data were converted to scores between 0 and 100, with a higher score indicating better performance for that item. 

Given the service conditions of ship lock gallery concrete, where failure is primarily controlled by high-speed sediment-laden flow abrasion and pressurized water penetration [28], as well as the high requirements for freeze–thaw resistance and crack resistance, the following weights were assigned to each performance indicator: an abrasion–erosion resistance of 30%, a crack resistance (jointly characterized by initial cracking time weight 2/3 and maximum crack width weight 1/3) of 30%, impermeability of 20%, and a freeze–thaw resistance of 20%. This weight distribution reflected the practical emphasis on durability requirements for hydraulic structures.

#### 3.7.2. Results Analysis and Optimal Decision

Based on the comprehensive scoring results in Table 7, range analysis of the comprehensive scores indicated that fiber content had a slightly greater influence (R = 45.9) than fiber length (R = 43.4). This suggested that, within the scope of this experiment, fiber content had a slightly more dominant influence on comprehensive durability performance. Judging from the mean values at each level, the comprehensive score was highest when the fiber content was 0.20% (K1 = 76.6) and when the fiber length was 12 mm (K2 = 82.9). Specifically, for the experimental groups, group 2 (content 0.20%, length 12 mm) achieved a high comprehensive score of 91.9. It performed excellently in all four indicators—crack resistance, impermeability, freeze–thaw resistance, and abrasion–erosion resistance—receiving perfect scores for crack resistance and impermeability, in particular.

It is worth noting that the optimal mix proportion derived from this stage of full factorial experiments (water-to-binder ratio 0.35, fiber content 0.2%, fiber length 12 mm) was completely consistent with the theoretical optimal combination determined through range analysis in Section 3.2. This, on the one hand, verified the reliability of the orthogonal experiment conclusions, while on the other hand, it indicated that this mix proportion was a robust choice for achieving the best balance between mechanical and durability performance, rather than a coincidental result under specific experimental conditions.

In summary, based on the comprehensive evaluation and range analysis of the full factorial experiments, and fully integrating mutual verification with the orthogonal experiment results, the optimal mix proportion for basalt fiber-reinforced hydraulic concrete was ultimately determined as: a water-to-binder ratio of 0.35, a fiber content of 0.20%, a fiber length of 12 mm.

## 4. Discussion

This chapter focuses on analyzing the performance enhancement mechanism of basalt fiber (BF)-reinforced concrete, the primary and secondary influence of key parameters, and their interactions based on the aforementioned test results. It is also compared with existing research to clarify the engineering value of this study.

### 4.1. Analysis of Performance Enhancement Mechanism

The results of this study clearly indicate that the performance enhancement of hydraulic concrete by basalt fiber (BF) is the result of synergistic effects across multiple scales and through various mechanisms. These mechanisms do not exist in isolation but constitute a complete enhancement system with stepwise transmission and mutual reinforcement [29].

#### 4.1.1. Nano–Micro-Scale

At the nano- and micro-scale, the performance of fiber-reinforced concrete is governed by two primary factors: the quality of the cement matrix and the quality of the fiber-matrix interfacial transition zone (ITZ). The water-to-binder ratio is a critical parameter that simultaneously regulates both [30].

On the one hand, reducing the water-to-binder ratio directly optimizes the matrix itself by decreasing the total porosity and reducing the number of harmful large pores, thereby forming a dense microstructure. This provides a robust anchoring foundation for the fibers. On the other hand, a lower water-to-binder ratio significantly weakens the “wall effect” near the fiber surface, promoting a denser ITZ with a reduced porosity, refined calcium hydroxide (CH) crystal size, and more uniform crystal distribution.

**Figure 9:**
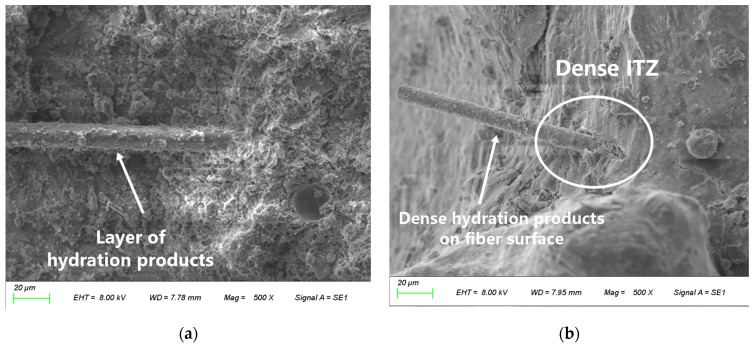
Interfacial hydration products and fiber–matrix bonding. (**a**) Hydration products densely wrapping the fiber surface; (**b**) dense interfacial transition zone (ITZ).

**Figure 10:**
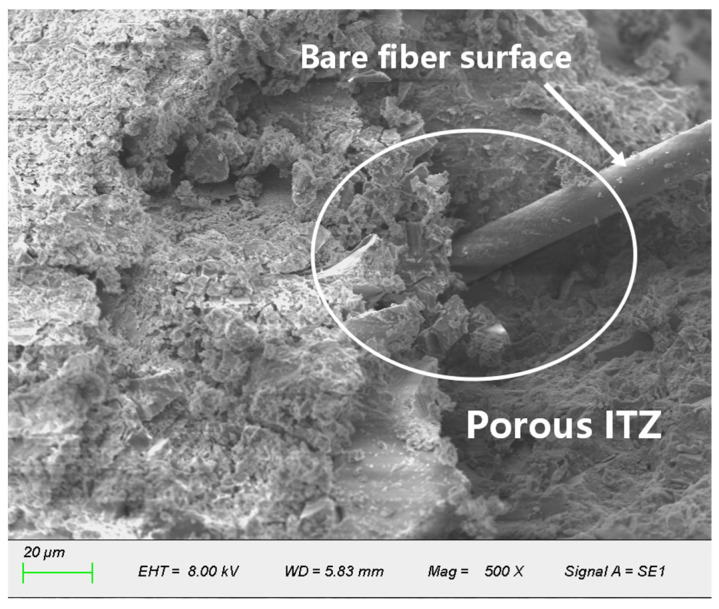
Loose interfacial transition zone under high water-to-binder ratio.

The low water-to-binder ratio of 0.35 employed in this study validates this mechanism (Figure 9 and Figure 10); that is, this mix proportion encourages hydration products (such as C-S-H gel) to densely wrap the fiber surface, forming an interfacial layer characterized by low porosity as well as high strength and toughness. The micromechanical properties of this dense ITZ structure (e.g., elastic modulus and hardness) are consequently enhanced.

At a more fundamental nano-scale level, this efficient stress transfer is further underpinned by enhanced chemical bonding. Silanol groups (-Si-OH) on the basalt fiber surface form stable -Si-O-Ca- covalent bridges with calcium ions in the C-S-H gel [31], which work synergistically with van der Waals forces and hydrogen-bonding networks. This combination of chemical anchoring and physical adsorption, together with the dense physical structure as described above, can collectively provide a complete foundation for achieving efficient multi-scale stress transfer and preventing ineffective fiber pull-out [32].

#### 4.1.2. Meso-Scale

**Figure 11:**
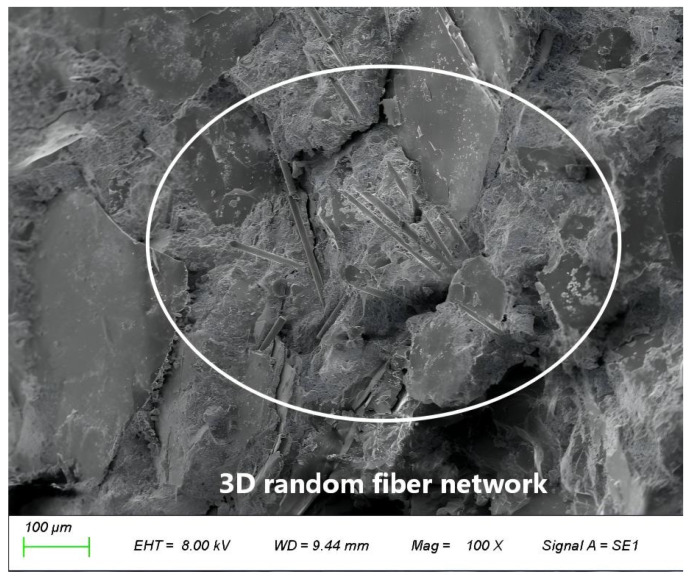
Uniform three-dimensional distribution of fibers under suitable mix proportions.

**Figure 12:**
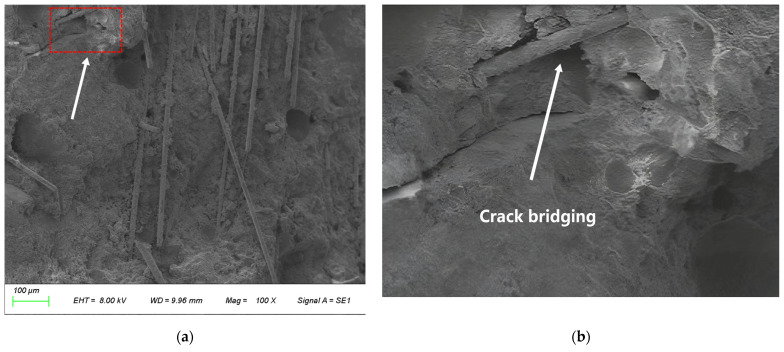
Microscopic morphology of basalt fibers bridging a micro-crack. (**a**) Microscopic morphology of the concrete matrix; (**b**) enlarged view of the red box area in (**a**) showing a basalt fibers bridging a micro-crack.

Fibers that are uniformly dispersed in the concrete form a three-dimensional randomly distributed network (Figure 11) [33]. As the primary function of this network, bridging can fundamentally reconstruct the mechanical behavior of concrete through the adaptive regulation of the entire damage evolution process:

(1) During the plastic and early hardening stages, this network can serves as a micro-reinforcement by effectively dispersing internal tensile stress induced by plastic shrinkage, temperature changes, or loading, thereby delaying the initiation of micro-cracks [34]. This directly aligns with the significant extension of cracking time in the ring test.

(2) After micro-cracks have been generated, fibers spanning the cracks exert a bridging effect (Figure 12) through their strong interfacial bond strength. This behavior not only constrains crack propagation but also triggers a fundamental transformation of the failure mode—dispersing concentrated, destructive wide cracks into numerous fine, harmless micro-crack systems, which significantly improves the material’s post-cracking toughness and energy dissipation capacity [35].

(3) The bridging and stress transfer effects of fibers not only directly improve tensile and flexural strength, but also indirectly enhance compressive strength and residual strength by restraining the lateral deformation of the matrix [36,37].

(4) This network optimizes the pore structure of the matrix through physical action, reduces connected pores, and divides some large pores into small, closed pores. Simultaneously, the dense fiber network greatly prolongs, bends, and blocks the migration paths of fluids and aggressive ions. Both impermeability and freeze–thaw resistance are fundamentally improved by this structural self-compacting effect [38].

**Figure 13:**
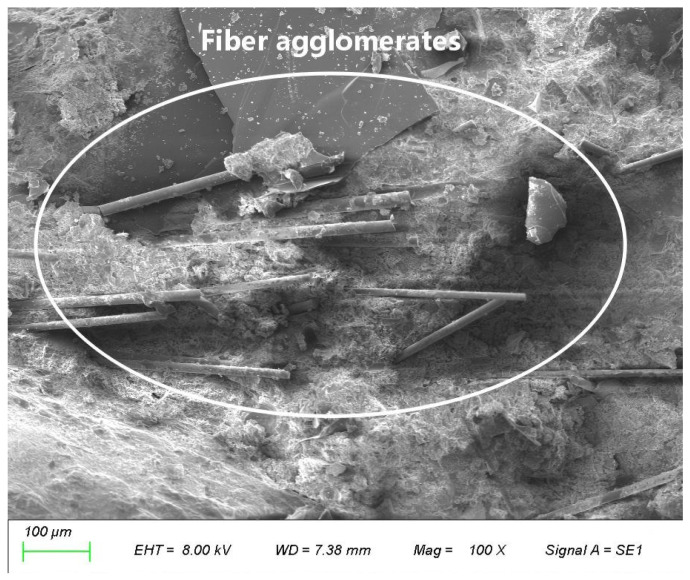
Fiber agglomeration induced by high fiber content.

The realization of these functions had been predicated in the uniform dispersion of fibers within the matrix. However, “dispersion” encompasses two distinct aspects: the uniformity of fiber spatial distribution and the randomness of fiber orientation. An ideal three-dimensional network requires both characteristics to ensure these isotropic material properties. Notably, multiple microscopic observations reveal that poor fiber dispersion (agglomeration) is often accompanied by a consistent local orientation (as shown in Figure 13). This implies that once uniformity has been compromised, orientation randomness is also lost [39].

Further analysis of the relationship between the fiber length and dispersibility reveals a significant interaction between these two factors. Under the premise of good dispersion, shorter fibers (6–9 mm), while exhibiting a high orientation randomness, struggle to fully realize their bridging effect due to an insufficient anchorage length. Conversely, longer fibers (15–18 mm) offer superior anchorage, but their larger aspect ratio makes them prone to an oriented alignment in the flow field—when dispersion is uniform, this manifests as enhanced orientation consistency in local regions, but when dispersion is poor, it directly exacerbates entanglement and agglomeration [40].

Consequently, the 12 mm fiber achieved an optimal comprehensive performance in this study. Essentially, this is because it reaches an ideal equilibrium in the ternary balance of “uniformity–randomness–anchorage length”, where its length is sufficient to ensure effective anchorage and bridging, while simultaneously maintaining a good dispersion and orientation randomness under conventional mixing processes.

#### 4.1.3. Macro-Scale

In the simulated high-velocity water flow abrasion environment of a ship lock gallery, the fiber enhancement mechanism manifests as a dynamic coupling of macro-scale “anchoring” and “energy dissipation.”

Anchoring Effect: The three-dimensional fiber network physically interweaves and entangles, mechanically locking the aggregates and mortar in the concrete and forming a composite material system with extremely strong integrity [41]. This effect inhibits the integral spalling of aggregates, transforming the failure mode from brittle spalling to progressive surface wear [42].

Bridging–Energy Dissipation Effect: Under dynamic impacts, fibers spanning the micro-cracks primarily dissipate energy through a progressive pull-out process from the matrix. During this process, their strong interfacial bond strength generates continuous friction, converting the concentrated impact kinetic energy into dispersed thermal energy. This mechanism, dominated by interfacial frictional energy dissipation, is the main reason for the substantial increase in the abrasion–erosion resistance strength.

In summary, the enhancement by basalt fiber is a multi-scale synergistic process, encompassing bonding at the nano-interface, stress transfer at the micro ITZ, and damage regulation by the meso-scale three-dimensional network, as well as ultimately manifesting as a comprehensive improvement in durability performance at the macro-scale.

#### 4.1.4. Limitations and Outlook on Microscopic Analysis

It should be noted that the microscopic mechanism analysis presented above is primarily based on SEM morphological observation, which is a qualitative characterization method. Systematic quantitative tests regarding the statistical distribution of fiber orientation, three-dimensional spatial distribution characteristics of agglomerates, and micromechanical properties of the ITZ (such as elastic modulus, hardness, and interfacial bond strength) have not been conducted in this study. Quantitative characterization of these microscopic features requires advanced techniques such as X-ray computed microtomography (X-CT) and nanoindentation, representing a direction for future in-depth investigation. Nevertheless, the consistency observed between microscopic morphology and macroscopic performance in this study provides valuable qualitative evidence for the reinforcement mechanism of basalt fibers and establishes an experimental foundation for subsequent quantitative research.

### 4.2. Parameter Balance of the Optimal Mix Proportion

Through single-factor tests, orthogonal experiments, and full factorial durability verification, this study has determined the optimal mix proportion as: a water-to-binder ratio of 0.35, a fiber content of 0.2%, a fiber length of 12 mm. This mix proportion represents a comprehensive outcome balancing high strength, high durability, and good workability.

#### 4.2.1. Water-to-Binder Ratio

The selection of the water-to-binder ratio requires a balance between ITZ quality and workability. The low water-to-binder ratio of 0.35 maximizes matrix strength and interfacial bonding efficiency while ensuring adequate workability (slump > 50 mm), providing an ideal foundation for fiber reinforcement [43].

Higher water-to-binder ratios (e.g., 0.45), while offering better workability, can lead to looser ITZ and substantially reduced fiber reinforcement efficiency, resulting in deteriorated comprehensive performance.

#### 4.2.2. Fiber Content

Fiber content exerts a decisive influence on performance by regulating the dispersion state and interface characteristics. The optimal level is 0.2%, at which fibers are uniformly distributed, forming an ideal three-dimensional support network and efficiently transferring stress through the dense ITZ. Figure 11 presents the typical microscopic morphology at 0.2% fiber content, showing fibers uniformly dispersed within the matrix and establishes the foundation for fully realizing the bridging and toughening effects [44].

**Figure 14:**
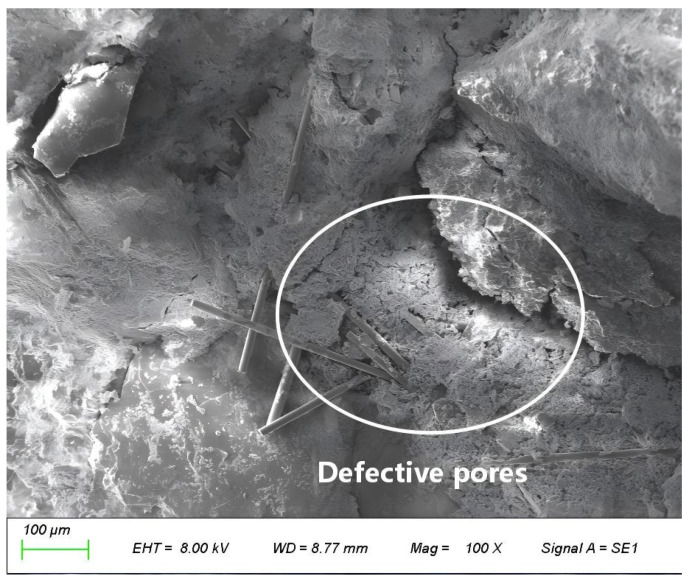
Defective pores formed around fiber agglomerates in concrete with 0.3% fiber content.

An appropriate amount of fiber can reduce bleeding and shrinkage cracks, lower porosity, and optimize the internal structure, all of which enhance mechanical properties through three aspects: crack inhibition, toughening, and reinforcement [33,41,43]. However, when the fiber content is too high (e.g., 0.4%), this enhancement effect weakens or even reverses into deterioration. Basalt fibers possess a large specific surface area and strong surface hydrophilicity; excessive incorporation readily leads to mutual entanglement and agglomeration during mixing, forming fiber bundles or clusters that cannot effectively transfer stress. SEM observations reveal that when the content increases to 0.4%, fibers exhibit significant agglomeration. In this study, the criterion for identifying “fiber agglomeration” is defined as multiple fibers intertwining and clustering to form macroscopically discernible fiber clusters, with the inter-fiber spacing within the cluster being less than the fiber diameter. These agglomerates not only act as internal stress concentration points, inducing premature failure, but also severely deteriorate workability, hindering paste flow and compaction [33]. Simultaneously, fiber agglomeration introduces numerous air bubbles, which forming defective pores around the clusters (Figure 14) and lead to an increased overall porosity and a higher proportion of harmful pores, thereby causing a comprehensive decline in compactness, strength, and durability [42].

#### 4.2.3. Fiber Length

Fiber length directly affects the balance between anchoring efficiency and dispersibility. Excessively short fibers (e.g., 9 mm) may be pulled out before being fully fractured due to insufficient embedment depth, failing to fully utilize their tensile performance and significantly limiting the bridging effect. Excessively long fibers (e.g., 15 mm) are more prone to entanglement and agglomeration during mixing, forming localized enrichment zones and weak points that cannot effectively transfer stress and instead become sources of stress concentration which promote early failure [10].

The full factorial experiment results (Table 7) demonstrate that at 0.2% fiber content, the comprehensive score of the 12 mm length group (91.9) is significantly higher than those of the 9 mm group (51.1) and the 15 mm group (86.8), confirming that the 12 mm length achieves an optimal balance between anchorage efficiency and dispersibility. This finding is consistent with the mechanistic analysis presented in Section 4.1.2.

The full factorial experiment further confirms the interaction between fiber content and length: at 0.2% content, increasing the length from 9 mm to 12 mm enhances bridging span and energy dissipation capacity; however, at 0.3% content, increasing the length to 15 mm drastically intensifies entanglement and leads to severe performance degradation [45].

#### 4.2.4. Comprehensive Trade-Off of Performance Objectives

Group C3 (0.3%, 12 mm) performed excellently in impermeability and controlling crack width, but its mechanical properties and initial cracking time had been slightly inferior to group C2. This reflects that while a higher content may further improve certain durability aspects (e.g., impermeability), it might negatively impact mechanical properties due to slight agglomeration. The final selection of group C2 as the optimal mix proportion reflects a higher pursuit of the robustness of comprehensive performance and long-term safe service for ship lock gallery concrete.

### 4.3. Innovative Contributions of This Study

This study, oriented towards the complex service environment of ship lock galleries, adopts a progressive research path of “single-factor preliminary screening → orthogonal optimization → full factorial verification,” forming innovative contributions at three levels: variable selection, mechanistic understanding, and engineering specificity.

1. This study reveals the dominant role of the water-to-binder ratio in the fiber-reinforced system and establishes a synergistic enhancement paradigm of “strong matrix–optimized interface–low fiber content”. Existing studies often fixed the water-to-binder ratio (typically ≥0.40) and only optimized fiber content and length [34]. This study incorporates the water-to-binder ratio as a core variable. Orthogonal experiments indicate that it is the most dominant factor affecting comprehensive performance (range R = 57.4), far exceeding fiber content (R = 46.8) and length (R = 11.4). The high-strength, dense matrix constructed by the 0.35 low water-to-binder ratio significantly weakens the ITZ “wall effect”, enabling the 0.2% low-content scheme to meet or exceed traditional high-content schemes in multiple indicators, such as abrasion–erosion resistance, impermeability, and freeze–thaw resistance. This aligns with the findings from studies on high-performance materials like ultra-high-performance concrete [39,46] and those exploring bond behavior in complex matrices [11,47].
2. This study further reveals the law that fiber reinforcement efficiency is positively correlated with matrix strength, providing a theoretical basis for the “matrix-dominated, fiber-synergistic” design concept. Through a cross-scale correlation analysis from nano-scale chemical bonding (-Si-O-Ca- covalent bridges) → micro-scale ITZ densification → meso-scale three-dimensional network bridging → macro-scale durability improvement, the intrinsic mechanism of the “strong matrix–optimized interface–low fiber content” paradigm is elucidated as follows. The low water-to-binder ratio optimizes the ITZ structure, improving the stress transfer efficiency of individual fibers, while the three-dimensional network achieves crack bridging and pore optimization, which ultimately translates into macroscopic performance improvements. The orthogonal experiment and the full factorial experiment jointly confirm that this synergistic effect of matrix–interface–fiber can make prioritizing matrix optimization (reducing the water-to-binder ratio) more effective than simply increasing fiber content—the water-to-binder ratio is the dominant factor for performance (R = 57.4), while the low 0.2% content achieves optimal comprehensive performance under the optimized matrix.
3. This study establishes a customized research system oriented towards the multi-objective service conditions of ship lock galleries. Existing studies have mostly targeted conventional structures such as road pavements and bridge decks [34,46], and systematic research on special hydraulic environments like ship lock galleries is still lacking. Ship lock gallery concrete simultaneously withstands the coupled effects of high-velocity sand-laden flow abrasion, penetration pressure, freeze–thaw cycles, and shrinkage cracking, and its failure mode is fundamentally different from that of conventional structures. The engineering specificity of this study is reflected in two aspects: First, the specificity of experimental design, with specially designed tests for abrasion–erosion resistance, impermeability, freeze–thaw resistance, and ring constraint cracking, comprehensively covering actual service conditions. Second, the specificity of the evaluation system. Based on the stress characteristic that ship lock gallery linings primarily bear circumferential tensile stress, a mechanical performance weighting system is established (flexural 40%, splitting tensile 30%, compressive 30%). Based on failure experience controlled by abrasion and permeation, a durability performance weighting system is also established (abrasion–erosion resistance 30%, crack resistance 30%, impermeability 20%, freeze–thaw resistance 20%). This research approach, which originated with engineering and has returned to engineering, translates enhancement principles into a systematically verified optimal mix proportion, providing an operable engineering solution for ship lock galleries.

### 4.4. Engineering Application Potential Beyond Ship Lock Galleries

Although this study focuses on ship lock galleries as a specific hydraulic structure, the basalt fiber reinforcement mechanisms and optimal mix proportion revealed herein are also relevant to other engineering structures that are subjected to similar complex loads and environmental actions.

In bridge and tunnel engineering, deck pavements and tunnel linings are subjected to long-term fatigue from vehicle loads, temperature variations, and shrinkage stresses, with crack control and durability requirements similar to those of ship lock galleries. The “strong matrix-optimized ITZ-low fiber content” synergistic enhancement model established in this study, together with the improved crack resistance and impermeability provided by basalt fibers, could effectively delay fatigue cracking in bridge decks and leakage in tunnel linings [9].

In marine and port engineering, structures such as wharves and breakwaters operate under harsh environments featuring dry–wet cycles and chloride attacks, with deterioration mechanisms comparable to those of ship lock galleries. The significant improvements in impermeability and freeze–thaw resistance achieved by using basalt fibers in this study could offer valuable references for extending the service lives of marine concrete structures [48,49].

In road and airport pavement engineering, heavy traffic loads and thermal stresses render pavement structures susceptible to reflective cracking and fatigue damage. The three-dimensional network reinforcement effect of basalt fibers and the enhancement of concrete toughness could effectively disperse stress and delay crack propagation, providing a new technical approach for improving pavement and road durability.

In summary, the “strong matrix-optimized ITZ-low fiber content” synergistic enhancement model, along with the positive correlation between fiber reinforcement effectiveness and matrix strength revealed in this study, can provide a theoretical basis and design references for fiber-reinforced concrete applications in the aforementioned engineering fields.

## 5. Conclusions

This study adopted a progressive research methodology combining single-factor tests, orthogonal experiments, and full factorial experiments to systematically investigate the performance of basalt fiber-reinforced hydraulic concrete under the complex service environments of ship lock galleries. The optimal mix proportion was determined to be: a water-to-binder ratio of 0.35, a fiber content of 0.2%, and a fiber length of 12 mm. This mix proportion synergistically enhanced the crack resistance, impermeability, freeze–thaw resistance, and abrasion–erosion resistance of concrete: the initial cracking time extended by 69.9%, the maximum crack width reduced by 40.5%; the relative permeability coefficient decreased to 1.04 × 10^−6^ cm/h; the relative dynamic elastic modulus remained at 73.0% after 100 freeze–thaw cycles; and the abrasion–erosion resistance strength increased by 25.7%.

The innovative contributions of this study were mainly reflected in: revealing the dominant role of water-to-binder ratios in the fiber reinforcement system, establishing a “strong matrix-optimized ITZ-low fiber content” synergistic enhancement paradigm; elucidating the positive correlation between fiber efficiency and matrix strength; and constructing a customized research framework for multi-objective service conditions of ship lock galleries.

These findings have not only provided systematically validated solutions for the durability design of ship lock galleries, but they have also offered a theoretical basis and design references for other structures that are subjected to complex loads and environmental actions, such as bridges, tunnels, and marine engineering.

However, this study had certain limitations. The durability tests followed standard laboratory methods such as SL/T 352-2020. Although these methods could effectively evaluate the basic properties of materials, they could not fully simulate the dynamic load coupling effects (e.g., high-velocity sediment-laden flow, wet–dry cycles, long-term synergistic effects of temperature and loads) that ship lock galleries can experience in real marine environments. Furthermore, the micro-mechanism analysis was primarily based on qualitative SEM observations and lacked a statistical analysis of fiber three-dimensional distribution characteristics (such as orientation, spacing, and spatial uniformity), making it difficult to quantitatively reveal the intrinsic relationship between the fiber network structure and its macroscopic performance. Future research should focus on the following areas: conducting long-term field exposure tests to establish correlation models between accelerated laboratory tests and real service performance; combining nanoindentation, X-CT scanning, and image analysis techniques to quantitatively characterize ITZ micromechanical properties and fiber three-dimensional distribution characteristics; and developing multi-scale numerical simulation methods to reveal the damage evolution laws of fiber-reinforced concrete under coupled complex loads and environmental actions.

## Figures and Tables

**Table 1:** Single-factor experimental scheme.

Factor	Level Gradient	Fixed Parameters
Water-to-binder ratio	0.30, 0.35, 0.40, 0.43, 0.45, 0.50	Content 0.2%, Length 12 mm
Fiber content	0, 0.10%, 0.20%, 0.30%, 0.40%, 0.50%	Water-to-binder ratio 0.40, Length 12 mm
Fiber length	3 mm, 6 mm, 9 mm, 12 mm, 18 mm, 24 mm	Water-to-binder ratio 0.40, Content 0.2%

**Table 2:** Factors and levels table.

Factor	Water-to-Binder Ratio	Fiber Content (%)	Fiber Length (mm)
Level 1	0.35	0.1	6
Level 2	0.40	0.2	9
Level 3	0.43	0.3	12
Level 4	0.45	0.4	18

**Table 3:** Results of orthogonal experiment.

Specimen No.	Water-to-Binder Ratio	Fiber Content	Fiber Length	Compressive Strength (MPa)	Splitting Tensile Strength (MPa)	Flexural Strength (MPa)	Comprehensive Index
1	1	1	1	44.33(85.3) ^1^	4.39(89.9)	6.03(85.9)	86.9
2	1	2	2	46.16(100.0)	4.52(98.7)	6.23(97.2)	98.5
3	1	3	3	45.87(97.7)	4.54(100.0)	6.28(100.0)	99.3
4	1	4	4	37.94(34.1)	3.72(45.0)	5.5(55.9)	46.1
5	2	1	2	41.66(63.9)	3.92(58.4)	5.5(55.9)	59.1
6	2	2	1	43.37(77.6)	3.99(63.1)	5.72(68.4)	69.6
7	2	3	4	40.93(58.1)	3.85(53.7)	5.77(71.2)	62.0
8	2	4	3	36.27(20.7)	3.45(26.8)	5.01(28.2)	25.5
9	3	1	3	40.85(57.4)	3.68(42.3)	5.31(45.2)	48.0
10	3	2	4	42.43(70.1)	3.82(51.7)	5.73(68.9)	64.1
11	3	3	1	40.25(52.6)	3.54(32.9)	5.23(40.7)	41.9
12	3	4	2	35.63(15.6)	3.23(12.1)	4.85(19.2)	16.0
13	4	1	4	39.2(44.2)	3.44(26.2)	5.1(33.3)	34.4
14	4	2	3	40.27(52.8)	3.49(29.5)	5.31(45.2)	42.8
15	4	3	2	38.24(36.5)	3.31(17.4)	4.85(19.2)	23.9
16	4	4	1	33.69(0.0)	3.05(0.0)	4.51(0.0)	0.0
K1	82.7	57.1	49.6				
K2	54.0	68.7	49.3				
K3	42.5	56.8	53.9				
K4	25.3	21.9	42.5				
R	57.4	46.8	11.4				

^1^ Values in parentheses are normalized percentage scores.

**Table 4:** Analysis of variance (ANOVA) results.

Indicator	Water-to-Binder Ratio	Fiber Content	Fiber Length
Compressive strength	** ^1^	**	
Splitting tensile strength	**	**	
Flexural strength	**	**	
Comprehensive index	**	**	

^1^ (*) F > F_0.05_(3,6) = 4.76, (**) F > F_0.01_(3,6) = 9.78. Fiber length showed no significance (F < 4.76) and is therefore not marked.

**Table 5:** Ring restraint cracking test results.

Group	Water-to-Binder Ratio	Fiber Content (%)	Fiber Length (mm)	Initial Cracking Time (h)	Maximum Crack Width After 24 h (mm)
JZ′	0.35	–	–	40.8	0.42
C1′	0.35	0.2	9	65.6	0.28
C2′	0.35	0.2	12	69.3	0.25
C3′	0.35	0.3	12	64.7	0.21

**Table 6:** Impermeability test results.

Group	Relative Permeability Height (cm)	Relative Permeability Coefficient Kr (cm/h)
JZ	4.29	1.41 × 10^−6^
C1	3.82	1.12 × 10^−6^
C2	3.68	1.04 × 10^−6^
C3	3.75	1.08 × 10^−6^

**Table 7:** Full factorial experiment durability test results.

Exp. No.	Fiber Content (%)	Fiber Length (mm)	Crack Resistance		Impermeability	Freeze–Thaw Resistance	Abrasion–Erosion Resistance	Comprehensive Score
			Initial Crack Time (h)	Max. Crack Width (mm)	Rel. Perm. Coeff. (×10^−6^ cm/h)	Relative Dynamic Elastic Modulus (%)	Abrasion Resistance Strength (h·m^2^/kg)	
1	0.2	9	65.6(71.3) ^1^	0.28(41.7)	1.12(57.9)	69.69(48.2)	6.68(38.3)	51.1
2	0.2	12	69.3(100.0)	0.25(66.7)	1.04(100.0)	72.96(100.0)	7.05(84)	91.9
3	0.2	15	67.2(83.7)	0.24(75)	1.07(84.2)	72.31(89.7)	7.12(92.6)	86.8
4	0.25	9	60.4(31)	0.25(66.7)	1.19(21.1)	68.94(36.3)	6.89(64.2)	43.6
5	0.25	12	66.7(79.8)	0.23(83.3)	1.05(94.7)	71.85(82.4)	7.15(96.3)	88.6
6	0.25	15	64.1(59.7)	0.22(91.7)	1.14(47.4)	71.32(74)	7.18(100)	75.4
7	0.3	9	57.5(8.5)	0.23(83.3)	1.21(10.5)	67.43(12.4)	6.62(30.9)	23.9
8	0.3	12	64.7(64.3)	0.21(100)	1.08(78.9)	71.78(81.3)	6.73(44.4)	68.2
9	0.3	15	56.4(0)	0.33(0)	1.23(0)	66.65(0)	6.37(0)	0.0
K1	76.6	39.5	-	-	-	-	-	-
K2	69.2	82.9	-	-	-	-	-	-
K3	30.7	54.1	-	-	-	-	-	-
R	45.9	43.4	-	-	-	-	-	-

^1^ Values in parentheses are normalized percentage scores.

## Data Availability

The original contributions presented in this study are included in the article. Further inquiries can be directed to the corresponding author.
